# Acute paraquat poisoning with sinus bradycardia: A case report

**DOI:** 10.3892/etm.2014.1944

**Published:** 2014-09-02

**Authors:** CHENGZHEN SONG, BAOTIAN KAN, GUANGCAI YU, XIANGDONG JIAN, JIERU WANG, JING SUN

**Affiliations:** Department of Poisoning and Occupational Diseases, Qilu Hospital of Shandong University, Jinan, Shandong 250012, P.R. China

**Keywords:** paraquat, poisoning, sinus bradycardia, treatment

## Abstract

Paraquat (PQ) is a highly toxic herbicide, which not only leads to acute organ damage, but also to a variety of complications. Patients with severe PQ-induced poisoning may succumb to multiple organ failure involving the circulatory and respiratory systems. Although numerous studies have been performed investigating PQ poisoning, cases of extreme bradycardia caused by acute PQ-induced poisoning are rare. In the present case report, a 59-year-old male who ingested PQ was admitted to the Department of Poisoning and Occupational Disease at Qilu Hospital of Shandong University (Jinan, China) after three days. The patient received treatment known as the ‘Qilu scheme’, which was established in the Department of Poisoning and Occupational Disease. However, the heart rate of the patient remained low following the administration of conventional medicines, until thyroid tablets were administered. To the best of our knowledge, cases of bradycardia following PQ poisoning are rare.

## Introduction

The herbicidal properties of paraquat (PQ) were first identified in the 1950s, with PQ first marketed as a therapeutic agent in 1962. Contact of paraquat with soil causes immediate absorption and complete loss of its activity (1); thus, PQ is widely used around the world in agriculture. Presently, PQ is the second highest-selling weed killer globally and is available in a 20% solution form that requires dilution prior to agricultural use (2). PQ is a highly toxic compound and PQ poisoning is difficult to treat clinically due to the lack of effective treatments. Patients with severe PQ-induced poisoning may succumb to multiple organ failure involving the circulatory and respiratory systems. Ingestion of ~30 ml of PQ usually leads to circulatory failure within two days, whereas patients who ingest less than one mouthful often survive the early phase of PQ poisoning (3). In China, the use of PQ has been virtually banned in the future (4). However, a number of PQ poisoning cases remain, the majority of which are suicide attempts. Numerous patients have been treated at the Qilu Hospital of Shandong University (Jinan, China); however, cases of extreme bradycardia caused by acute PQ poisoning have rarely been observed. In the current study, a case of extreme bradycardia as a result of acute PQ poisoning is presented.

## Case report

A 59-year-old male was admitted to the Department of Poisoning and Occupational Disease at the Qilu Hospital of Shandong University three days following an attempted suicide by PQ poisoning via oral admission. The patient became intoxicated, and ingested ~50 ml PQ at 13:20 on August 17, 2013. Minutes later, the patient was found by his family and was found to have vomited. On the third day the patient was transferred to our hospital for further treatment. The patient was lethargic on the way to the hospital, following administration of an emetic.

**Figure 1:**
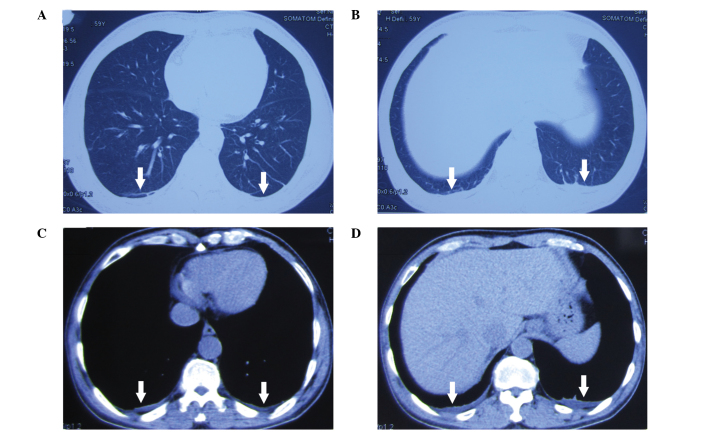
Chest computed tomography scan at day 3 following paraquat poisoning.

The patient was treated at the local hospital for alcohol consumption, without gastolavage and hemoperfusion. On the second day, the patient admitted to ingesting PQ; thus, was subsequently treated with steroid pulse therapy. The patient had no previous history of disease. During physical examination on admission to our local hospital, the patient was conscious with a blood pressure of 138/82 mmHg, a pulse rate of 61 bpm, a respiratory rate of 18 breaths/min, a blood oxygen saturation level of 98% and pharyngeal swelling was observed. The concentration of PQ in the urea was ~30 μg/ml. Analysis of the blood revealed a white blood cell count of 16.98×10^9^/l, a serum creatine level of 141 μmol/l and a blood sugar level of 19.8 mmol/l. In addition, effusion shadows and an unclear boundary in the double lower lobe were observed in the lungs following a computed tomography scan (Fig. 1).

**Figure 2:**
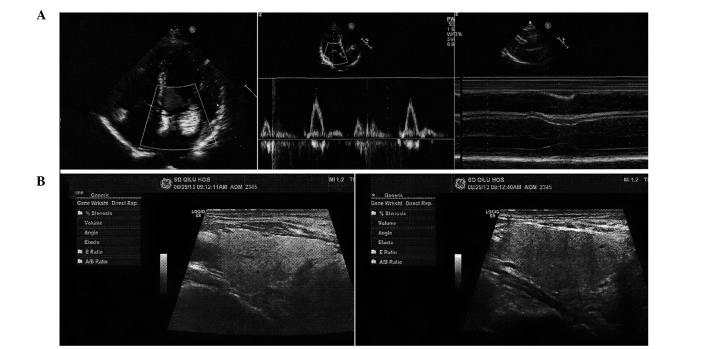
(A) An ultrasonic cardiogram revealed enlargement of the right atrium, widening of the ascending aorta, moderate mitral valve regurgitation, light medium tricuspid regurgitation and mild pulmonary valve regurgitation. (B) Ultrasound scans show that the thyroid gland was normal in size; however, a cyst (0.15 × 0.13 cm) was observed in the left lower pole.

**Figure 3:**
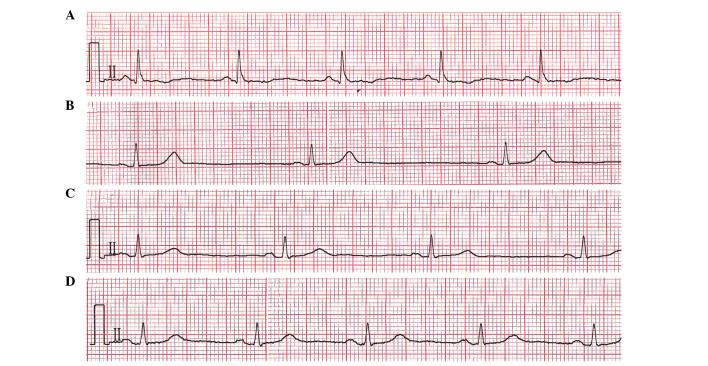
Changes in the electrocardiogram of a patient following paraquat poisoning at days (A) 2, (B) 5, (C) 7 and (D) 14.

The patient was administered 1,000 mg methylprednisolone, which was gradually reduced according to the health condition. Furthermore, whole gastrointestinal lavage, protection of the gastrointestinal mucosa, free radical scavenging, protection of the liver and kidneys, myocardial nutrition and maintenance of the water and electrolyte balance were applied via the administration of the ‘Qilu scheme’ (Department of Poisoning and Occupational Diseases) (5). However, on August 23, bradycardia occurred, and the patient had a pulse of 35 bpm. The heart rate of the patient gradually increased to 53 bpm following an intramuscular injection of atropine, but decreased to 40 bpm after 3 h. The following day, isoprenaline was administered continuously via an intravenous drip, while 0.6 mg atropine was administered orally three times a day. However, the heart rate remained low at 35–45 bpm. An ultrasonic cardiogram revealed enlargement of the right atrium, widening of the ascending aorta, moderate mitral valve regurgitation, light-medium tricuspid regurgitation and mild pulmonary valve regurgitation (Fig. 2A). Furthermore, the ultrasound revealed that the thyroid gland was normal in size; however, a cyst (0.15 × 0.13 cm) was observed in the left lower pole (Fig. 2B). Additional biochemical analyses revealed that the creatine kinase (CK) level was 50 IU/l, the CK-MB level was 0.9 ng/ml, the cardiac troponin I level was 0 ng/ml, the triglyceride (TG) level was 2.59 mmol/l, the free triiodothyronine (T_3_) level was 1.18 pg/ml, the free thyroxine (T_4_) level was 10.90 pmol/l and the thyroid stimulating hormone (TSH) level was 2.550 μIU/ml. Atropine and isopropyl adrenaline treatments were stopped and thyroid tablets were administered (80 mg per day). The heart rate increased to 50–55 bpm and was steady. Changes in the electrocardiograms are shown in Fig. 3. Following the treatment, the main examination index returned to normal with mild obstructive ventilation dysfunction observed in lung function. The patient was discharged from hospital, but received a follow-up examination one month later. The heart rate of the patient was 63 bpm and the level of N-terminal pro-brain natriuretic peptide was 22.17 pg/ml, the cortisol level was 15.75 μg/dl, the thyroglobulin antibody level was 18.26 IU/ml and the antithyroid peroxidase antibodies level was 14.08 IU/ml. With the exception of a degree of pulmonary fibrosis, the other examinations, including blood routine examinations and blood biochemical tests, were found to be normal. A written informed patient consent was obtained for this study.

## Discussion

PQ is a herbicide that is highly toxic to humans. Following the rapid oral absorption of PQ, a peak level is reached at 60–90 min following ingestion regardless of the plasma PQ levels (6). Misdiagnosis with alcohol poisoning during the early stages may result in patients not receiving gastric lavage and hemoperfusion, and as a consequence, the majority of the PQ is absorbed into the body. In the present study, the pulse rate of the patient decreased to 29 bpm from 61 bpm on admission to hospital. Isoprenaline was administered continuously via an intravenous drip, while atropine was administered orally; however, the condition of the patient did not improve. Treatment with atropine and isopropyl adrenaline was terminated and thyroid tablets were administered. The heart rate increased to 50–55 bpm and was steady. To the best of our knowledge, cases of bradycardia following PQ poisoning are rare. It was hypothesized that the following factors contributed to the decrease in the heart rate. Firstly, PQ poisoning may lead to toxic myocarditis (7) or dysfunction of the sinus node. Secondly, eating disorders and serious nutritional shortfalls may decrease the transport of T_4_ and T_3_ into tissues (8). Furthermore, in patients with existing heart conditions, low T_3_ levels may lower the neuroendocrine profile and ventricular performance (9). Finally, acute treatment with large doses of glucocorticoids results in a reduction of basal TSH levels. Pfister *et al* (10) demonstrated that free T_3_ and low-T_3_ syndrome are predictors of mortality, which are independent from other known cardiovascular risk parameters.

In conclusion, low levels of T_3_ are usually produced under the influence of a number of factors. When thyroid hormone levels are too low, a decrease in target organ function commonly occurs, and in particular, the heart rate may decrease. In addition, individuals with heart disease are more susceptible to being affected by levels of thyroid hormones. However, a number of other possible causes may lead to heart rate reduction; thus, further investigation is required.

## References

[1] Bullivant CM (1966). Accidental poisoning by paraquat: Report of two cases in man. Br Med J.

[2] Arts J, Schuit G, Schipper A, Kleij van der B (2006). A case report of PQ poisoning. Eur J Hosp Pharm.

[3] Bertram A, Haenel SS, Hadem J, Hoeper MM (2013). Tissue concentration of PQ on day 32 after intoxication and failed bridge to transplantation by extracorporeal membrane oxygenation therapy. BMC Pharmacol Toxicol.

[4] Yin Y, Guo X, Zhang SL, Sun CY (2013). Analysis of paraquat intoxication epidemic (2002–2011) within China. Biomed Environ Sci.

[5] Jian X, Zhang H, Sui H, Guo G (2014). Qilu Scheme of PQ poisoning treatment. Chinese Journal of Industrial Medicine.

[6] Kang MS, Gil HW, Yang JO, Lee EY, Hong SY (2009). Comparison between kidney and hemoperfusion for paraquat elimination. J Korean Med Sci.

[7] Magnani JW, Dec GW (2006). Myocarditis current trends in diagnosis and treatment. Circulation.

[8] Van der Heyden JT, Docter R, Van Toor H, Wilson JH (1986). Effects of caloric deprivation on thyroid hormone tissue uptake and generation of low-T3 syndrome. Am J Physiol.

[9] Pingitore A, Galli E, Barison A, Lervasi A (2008). Acute effects of triiodothyronine (T3) replacement therapy in patients with chronic heart failure and low-T3 syndrome: a randomized, placebo-controlled study. J Clin Endocrinol Metab.

[10] Pfister R, Strack N, Wielckens K, Malchau G (2010). The relationship and prognostic impact of low-T3 syndrome and NT-pro-BNP in cardiovascular patients. Int J Cardiol.

